# Fluorescent proteins as tools to aid protein production

**DOI:** 10.1186/1475-2859-4-12

**Published:** 2005-04-25

**Authors:** Wei Wen Su

**Affiliations:** 1 Department of Molecular Biosciences and Bioengineering, University of Hawaii at Manoa, Hawaii 96822, USA

## Abstract

Fluorescent proteins are genetically encoded, highly versatile reporters useful for monitoring various aspects of recombinant protein production. In addition to the widely popular green fluorescent protein (GFP) from *Aequorea victoria*, a variety of other fluorescent proteins have been discovered that display a wide range of spectral properties. Synthetic variants have also been developed to overcome limitations associated with their wild-type counterparts. Having a large repertoire of fluorescent proteins with diverse traits opens new opportunities for rapid monitoring and optimization of recombinant protein production.

## Review

### Introduction

**Figure 1:**
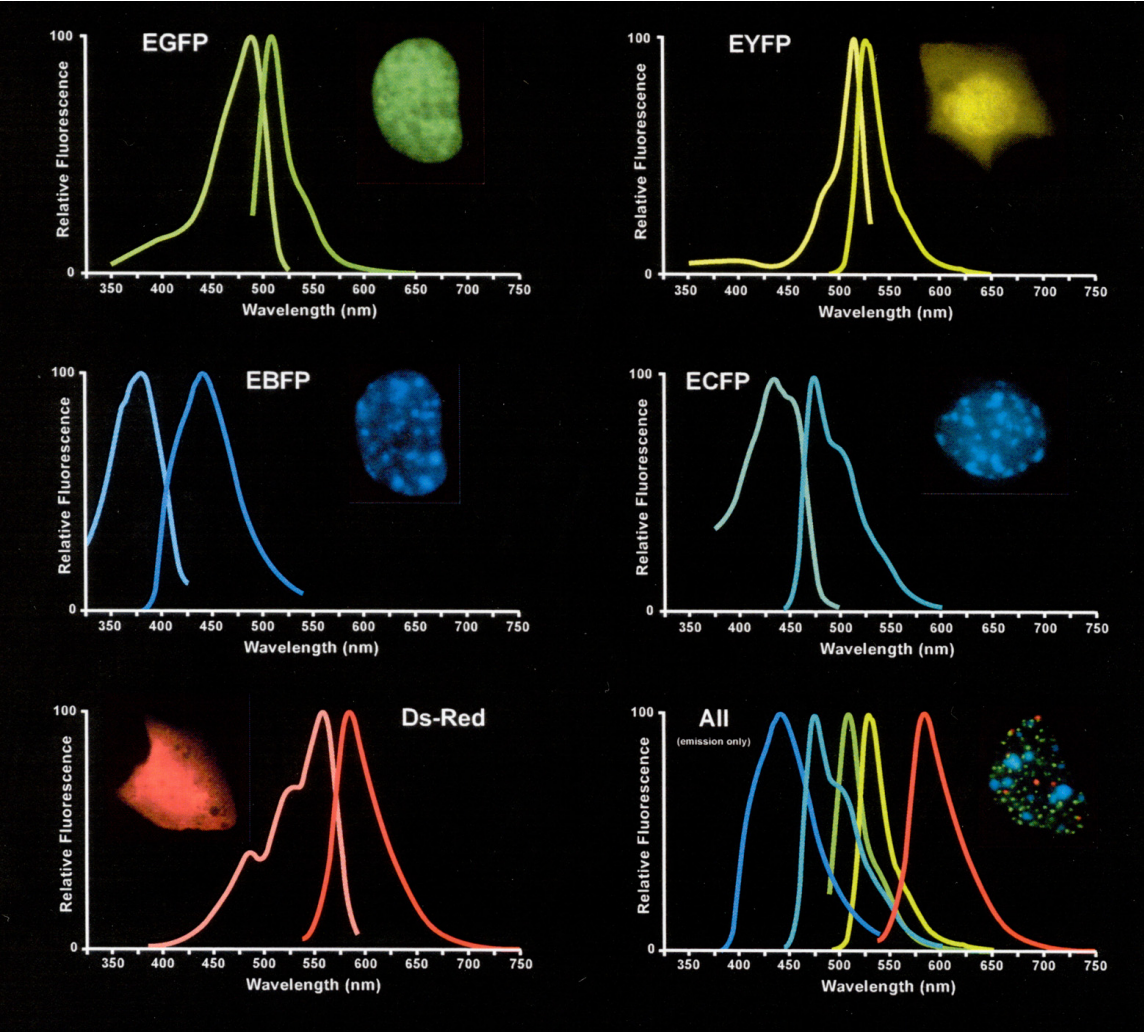
Fluorescence spectra of EGFP variants and DsRed (reproduced with the permission of [5]).

**Figure 2:**
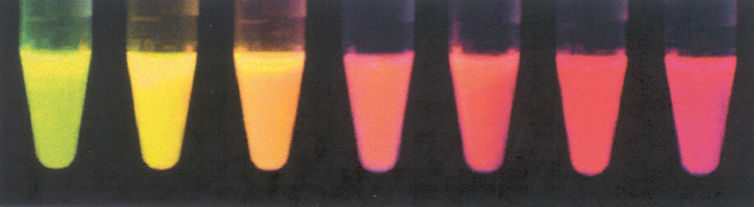
Fluorescence of DsRed variants developed by Shaner et al [6] (from left to right, mHoneydew, mBanana, mOrange, tdTomato, mTangerine, mStrawberry, mCherry; reproduced with the permission of [6]; refer to the same reference for details).

Expression of recombinant proteins from a variety of host organisms is now a common practice. However, production of properly folded proteins with high yield and purity may not always be achieved. Issues such as folding, solubility, protein stability, transcription and translation efficiency, posttranslational processing, secretion, metabolic burden and other stress responses resulting from recombinant protein production, as well as protein purification, need to be addressed in order to obtain biologically active recombinant proteins with high purity and yield [1]. In this regard, genetically encoded fluorescent reporters provide ample new opportunities to better tackle these issues. Since the demonstration of the *Aequorea victoria *green fluorescent protein (GFP) as a versatile reporter [2], several additional GFP-like fluorescent proteins with various colors have been discovered and their genes cloned [3]. Synthetic fluorescent protein variants have also been developed, exhibiting traits distinct from their wild-type counterparts. The properties of selected fluorescent protein variants derived from the *A. victoria *GFP and the *Discosoma *red fluorescent protein (DsRed) [4] are summarized in Table 1. Fluorescence spectra of enhanced GFP variants along with DsRed are shown in Figure 1[5], and fluorescence of purified protein variants derived from DsRed [6] are shown in Figure 2. Readers are referred to the work of Labas et al. [7] for information of additional fluorescent proteins. These GFP-like proteins each has its own unique properties, while sharing common structural, biochemical and photophysical characteristics [3]. GFP-like proteins are relatively small (25–30 kDa) and their fluorescence mechanism is self-contained, requiring no cofactors. These unique properties make GFP-like proteins very attractive tools in non-invasive biological monitoring applications. As a tool to improve recombinant protein production, fluorescent proteins can be used to monitor the protein product or the cellular processes relevant to recombinant protein production.

### Monitoring protein production, secretion, and culture growth

Fluorescent proteins are commonly used as a reporter for a protein of interest, normally by tagging the fluorescent protein reporter to the protein of interest *via *genetic fusion. Functional fusion of *Aequorea *GFP to a broad range of protein partners at either N- or C- terminus has been reported, and a direct quantitative correlation between the GFP fluorescence intensity and the titer or even the functional activity of the fusion partner can often be established [8,9]. To minimize potential interference by the GFP tag on its fusion partner, it is desirable and sometimes necessary to incorporate a peptide linker to allow sufficient spatial separation of the two protein moieties to assure fusion protein stability and functionality. Flexible linkers lacking large bulky hydrophobic residues (*e.g. *GSAGSAAGSGEF [10]) are commonly used, while hydrophilic helix-forming linker peptides have been reported to be superior to flexible linkers in some cases [11]. To allow removal of the GFP tag, an enzymatic cleavage site (*e.g. *enterokinase or Factor Xa cleavage sites) can be engineered into the linker. It is preferred to splice the GFP/linker to the N-terminus of the target protein, provided such fusion does not impair the target protein function and stability. With the majority of the enzymes commonly used for tag removal, this fusion orientation enables elimination of the tag without leaving extraneous amino acid residues on the target protein after cleavage. Alternatively, chemical cleavage based on cyanogen bromide, formic acid, or hydroxylamine may be considered, provided the target proteins are not susceptible to cutting by these chemical agents. Further information of tag removal can be found in a comprehensive review by Hearn and Acosta [12]. In addition to tandem fusion, insertional fusion (*i.e. *by inserting the protein of interest into GFP or *vise versa*) may also be feasible [13]. Recombinant protein production can be monitored non-invasively, *in situ*, and almost in real time, by monitoring culture fluorescence using on-line optical sensors [14,15]. This information is useful in determining the optimal product harvest time to avoid product degradation [16] and to devise process control strategies to optimize culture/operating conditions to improve recombinant protein production [8,17]. GFP has also been used to monitor recombinant virus titers in cell cultures [18], and cell density in microbial [15], animal [19], and plant cell cultures [8]; the cell growth information can be used, in turn, to optimize the culture process for improved recombinant protein production (*e.g. *by optimizing the feeding profiles of the limiting nutrient or the promoter inducer, or by determining the optimal product harvest time [8,17]). Additionally, GFP-fusion coupled with flow cytometric analysis is useful for profiling recombinant protein expression among different cell subpopulations, and selection of high-producing cells [9].

GFP-fusion can also be used for monitoring protein secretion and other subcellular protein localization and trafficking events. In the event direct GFP fusion hampers protein secretion, alternative protein fusion strategies may be sought. One plausible approach is to express GFP and a protein of interest as a cleavable chimeric polyprotein. By targeting the polyprotein to the secretory pathway, the target protein and the GFP tag may become separated and secreted as individual proteins. Feasibility of such approach has been demonstrated in fungi and plant cells for the successful *in-vivo *cleavage of an glucoamylase-interleukin-6 fusion protein [20] and a fusion antimicrobial polyprotein [21], respectively. Cellular processing in plant cells of a polyprotein that consists of DsRed-GFP fusion linked by a Kex2 cleavage sequence [22] is currently being investigated in the author's laboratory. In another possible approach, one may express the target gene and GFP in a dicistronic vector by incorporating an internal ribosome entry site (IRES) [23]. One additional possibility would be to express target protein and GFP as separate genes, but from the identical promoter. If a constant ratio between GFP fluorescence and target protein concentration could be established, the independent GFP could be used for fluorescent monitoring. Fluorescent protein reporters can also be used to probe protein-protein interactions that regulate the protein secretion process [24], potentially leading to development of molecular strategies that improve recombinant protein secretion.

### Monitoring protein purification

The fact that GFP fluorescence is readily detectable makes it a very attractive tool for optimizing purification of recombinant proteins. Poppenborg et al. [25] optimized immobilized metal affinity separation of a histidine-rich protein tagged with GFP by tracking the fluorescence of the fusion protein. Since GFP is a highly hydrophobic protein, recovery of GFP-fusion proteins can be facilitated by using hydrophobic interaction chromatography (HIC) [26]. GFP has also been engineered to allow affinity purification. Paramban et al [27] developed a chimeric GFP tag having an internal hexa-histidine sequence. Such a GFP tag allows efficient purification of GFP-fusion proteins based on immobilized metal affinity separation, as well as maximum flexibility for protein or peptide fusions since both termini of the GFP are available.

### Monitoring protein folding

Correct folding is one of the key challenges in recombinant protein production using simple hosts like *Escherichia coli*. GFP has been proposed as a folding reporter. By fusing GFP to a panel of proteins, Waldo *et al *[10] demonstrated that display of GFP fluorescence in the *E. coli *colonies expressing the fusion proteins indicated proper folding of GFP's fusion partner. De Marco [28] however cautioned that observation of GFP fluorescence from the fusion protein may not guarantee the fusion partner have reached its native structure.

Recently, Waldo and coworkers [29] reported a novel split-GFP system that consists of a small (GFP β strand 11; GFP 11) and a complementary large fragment (GFP β strand 1–10; GFP 1–10). Neither fragment by itself displays fluorescence, but GFP fluorescence emerges upon self-association of the two complementary fragments. These researchers demonstrated that by tagging target proteins with the GFP-11 tag, the solubility of the target proteins can be checked by mixing with the GFP 1–10 fragment in *vitro *or by co-expressing the GFP 1–10 fragment *in vivo*. Appearance of GFP fluorescence suggests the target protein is soluble. This split-GFP may also be useful for detecting protein-protein interaction *in vivo*, similar to the luciferase fragment complementation systems [30,31].

### Monitoring cellular responses

In addition to direct monitoring of protein expression, processing, and secretion, fluorescent proteins can also be used to monitor cellular events that are directly or indirectly related to recombinant protein production. For instance, FRET (fluorescence resonance energy transfer)-based GFP sensors have been developed to detect proteolysis *in vivo*. FRET-based GFP sensors have also been developed for measuring intracellular concentration of nitric oxide, calcium, cAMP, zinc, activation of G protein-coupled receptor (GPCR), and PKA-mediated phosphorylation [9]. Redox sensitive GFP variants have been developed for monitoring the cellular oxidative states, which strongly affect protein folding. In addition, intracellular pH can be measured using GFP. Metabolic stresses induced by recombinant protein over-expression may also be monitored *in vivo *using GFP linked to stress-induced promoters as a reporter.

### Optical sensing of culture GFP fluorescence

The presence of cell aggregates, debris, and other light absorbing/scattering compounds in the culture medium contributes to the "inner filter effect" (IFE) that could distort the optical measurement of culture GFP fluorescence. Real-time compensation of IFE in monitoring cultures expressing GFP-fusion proteins typically involves establishing a mathematical model to link the IFE to cell density, and to use an on-line laser turbidity sensor to report the biomass density needed in the calculation of the IFE [32]. An obvious drawback of such an approach is the requirement of a turbidity sensor in addition to the optical sensor for monitoring culture fluorescence. We recently developed a technique that allows real-time compensation of IFE during on-line monitoring of culture GFP fluorescence, without the need for an additional biomass sensor [33]. This was achieved by developing a model-based state observer, using the extended Kalman filter (EKF) and on-line measurement of GFP culture fluorescence using an optical light-rod sensor.

### Applications involving multiple fluorescent protein variants

Given the many facets of its applications, multiple fluorescent protein reporters could potentially be used in parallel for multi-color *in vivo *sensing. For instance, GFP may be used to tag the recombinant protein product, while a red fluorescent protein could be linked to a stress-responsive promoter such as the heat-shock promoter *groEL *to monitor stress induced by recombinant protein over-expression in *E. coli *[34]. Having a large repertoire of fluorescent proteins with diverse spectral properties is also necessary for multiplex FRET-based sensing applications. Through both structure-based modification and evolutionary methods for protein engineering, several robust variants of the *Aequorea *GFP have been created with blue, cyan, and yellow colors [35]. In a recent paper by Shaner et al [6], development of a panel of novel monomeric red, orange and yellow fluorescent proteins derived from the *Discosoma *DsRed was reported. These new variants also show additional traits that are useful for monitoring recombinant protein production.

### Monomeric fluorescent protein variants

Wild type DsRed is an obligate tetramer. When fused to a protein of interest, the fusion protein often forms aggregates, hampering normal localization, trafficking and protein-protein interactions of the protein of interest. A monomeric DsRed variant, called mRFP1, was developed by disrupting each subunit interface *via *insertion of arginines, and then using directed evolution to accelerate chromophore maturation and to restore fluorescence, which takes 33 substitutions [36]. mRFP1 was further improved by subjecting to additional rounds of directed evolution [6]. The resulting eight variants display corresponding emission peaks ranging from 537 to 610 nm. Some of these new variants also show better tolerance to N- and C- terminal fusions, higher extinction coefficients, quantum yields, and photostability, though no single variant has acquired all the desirable traits [6]. Since GFP is known to have high tolerance to either N- or C- terminal fusions, and DsRed and GFP share similar structures, Shaner et al [6] engineered GFP-type termini into mRFP1, rendering improved tolerance to protein fusion in the new variant. Among the new monomeric variants, mCherry (excitation at 587 nm, emission at 610 nm) is the most red-shifted, and has the best photostability, fastest maturation (15 min) and excellent pH resistance and tolerance to N-terminal fusions. The mOrange variant (excitation at 548 nm, emission at 562 nm) has high extinction coefficient and quantum yield, and is shown to be a superior FRET acceptor for GFP variants.

## Conclusion

The repertoire of fluorescent protein variants has continued to expand, and is now covering almost the entire color spectrum. With the advances in directed evolution techniques [37], each of these new proteins is likely to be further improved. The availability of improved multicolor fluorescent protein reporters will undoubtedly lead to development of innovative techniques that enable more effective multiplex cellular sensing, and allow more efficient on-line optimization of recombinant protein production.

## Figures and Tables

**Table 1:** Properties of selected fluorescent proteins

**Fluorescent Protein**	**Excitation Peak (nm)**	**Emission Peak (nm)**	**Extinction Coefficient (M^-1 ^cm^-1^)**	**Fluorescence Quantum Yield**	**Reference**
EBFP	383	445	31,000	0.25	[5]
ECFP	434	477	26,000	0.40	[5]
Cerulean CFP	433	475	43,000	0.62	[38]
EGFP	489	508	55,000	0.60	[5]
EYFP	514	527	84,000	0.61	[5]
Venus YFP	515	528	92,200	0.57	[39]
Citrine YFP	516	529	77,000	0.76	[40]
DsRed	558	583	75,000	0.79	[6]
mRFP1	584	607	50,000	0.25	[6]
mHoneydew	487/504	537/562	17,000	0.12	[6]
mBanana	540	553	6,000	0.70	[6]
mOrange	548	562	71,000	0.69	[6]
mTangerine	568	585	38,000	0.30	[6]
mStrawberry	574	596	90,000	0.29	[6]
mCherry	587	610	72,000	0.22	[6]
